# Direct Medical Costs of Parkinson’s Disease in Southern China: A Cross-Sectional Study Based on Health Insurance Claims Data in Guangzhou City

**DOI:** 10.3390/ijerph19063238

**Published:** 2022-03-09

**Authors:** Hui Zhang, Wenjing Zhou, Donglan Zhang

**Affiliations:** 1 School of Public Health, Sun Yat-sen University, No. 74, Zhongshan 2nd Road, Guangzhou 510080, China; zhouwj55@mail2.sysu.edu.cn; 2 Division of Health Services Research, New York University Long Island School of Medicine, Mineola, NY 11501, USA; donglan.zhang@nyulangone.org

**Keywords:** Parkinson’s disease, direct medical costs, health insurance, China

## Abstract

Background: Parkinson’s disease (PD) is the second most common neurodegenerative disorder. This study aims to evaluate the direct medical costs of patients with PD using a large sample from an entire city and to identity the potential factors correlating with their inpatient costs in Guangzhou City, Southern China. Methods: This retrospective cross-sectional study uses data obtained from the Urban Employee-based Basic Medical Insurance (UEBMI) and the Urban Resident-based Basic Medical Insurance (URBMI) administrative claims databases in Guangzhou City from 2008 to 2012. The total sample was comprised of 2660 patients with PD. Costs were evaluated for the total sample and by types of insurance. The composition of costs was compared between the UEBMI and URBMI subgroups. The extended estimating-equations model was applied to identify the potential impact factors influencing the inpatient costs. Results: The direct medical costs per patient with PD were CNY 14,514.9 (USD 2299.4) in 2012, consisting of inpatient costs of CNY 13,551.4 and outpatient costs of CNY 963.5. The medication costs accounted for the largest part (50.3%). The inpatient costs of PD patients under the UEBMI scheme (CNY 13,651.0) were significantly higher than those of patients in the URBMI subgroup (CNY 12,402.2) (*p* < 0.05). The proportion of out-of-pocket spending out of inpatient and outpatient costs for UEBMI beneficiaries (24.3% and 56.1%) was much lower than that for patients under the URBMI scheme (47.9% and 76.2%). The regression analysis suggested that types of insurance, age, hospital levels, length of stay (LOS) and comorbidities were significantly correlated with the inpatient costs of patients with PD. Conclusions: The direct medical costs of patients with PD in China were high compared to the GDP per capita in Guangzhou City and different between the two evaluated types of insurance. Patients with the UEBMI scheme, of older age, with comorbidities, staying in tertiary hospitals and with longer LOS had significantly higher inpatient costs. Thus, policymakers need to reduce the gaps between the two urban insurance schemes in benefit levels, provide support for the development of a comprehensive long-term care insurance system and promote the use of telemedicine in China.

## 1. Introduction

With a rapidly aging international population, Parkinson’s disease (PD) is becoming a worldwide public health problem and an economic burden [1,2]. Currently, PD is the second most common neurodegenerative disorder after Alzheimer’s disease and the most prevalent movement disorder [3]. Moreover, it is reported that PD maintains the highest growth in prevalence among all neurological disorders [4]. PD is characterized by its cardinal motor alterations (bradykinesia, resting tremors, rigidity and gait impairment) correlated to a loss of dopaminergic neurons in the substantial nigra, and also has other heterogeneous motor and nonmotor features [5]. A clinical diagnosis of PD is based on the presence of classical motor symptoms and additional supportive criterion [6]. Generally, PD is classified into two types: Idiopathic Parkinson’s disease (IPD) and Secondary Parkinsonism (SP) [7]. Now, it is widely recognized that the prevalence of SP is approaching to that of IPD, further increasing concerns [8].

According to the Global Burden of Disease Study, there were approximately 6.1 million people affected by PD globally in 2016 [9]. Patients in China were major contributors, with the number of people with PD amounting to 3.62 million in 2015 [10]. In China, the overall number of patients affected by PD will increase to 5 million by 2030, accounting for 50% of worldwide PD patients [11]. A meta-analysis of the worldwide data estimated the overall prevalence of PD to be 315 cases per 100,000 people [95% confidence interval (CI) = 113–873] [12]. In China, the prevalence of PD for people aged 60 years and older is 1.37% (95% CI 1.02–1.73) [10]. It was forecasted that the prevalence of PD would increase substantially in the coming future, especially in China [13].

PD affects patients’ health-related quality of life and imposes a heavy economic burden on individuals, healthcare systems and societies [14]. For example, the economic burden attributable to PD in the United States was USD 51.9 billion in 2017, and this figure was predicted to exceed USD 79 billion by 2037 [15]. In China, the annual expenditures on PD accounted for nearly 20% of gross domestic product (GDP) per capita in 2015 [16].

To enhance the level of financial protection and improve access to healthcare, China launched the Urban Employee-based Basic Medical Insurance (UEBMI) and the Urban Resident-based Basic Medical Insurance (URBMI) schemes [17], which were the two main social health insurance schemes administered by the Chinese government for urban residents only [17]. There are remarkable differences between UEBMI and URBMI in eligible population, financing sources and benefit packages [18]. The UEBMI scheme offers coverage to the employed urban workers, whereas those unemployed urban residents are enrolled under the URBMI scheme [17]. As for the financing sources, the premium of UEBMI comes from the contributions of both employers and employees, while the URBMI scheme is based on individual payments and government subsidies [19]. People under the UEBMI scheme have a more comprehensive coverage of health services and a higher reimbursement rate than those under the scheme of URBMI [19]. Therefore, it would be helpful for policymakers to obtain information on the direct medical costs of PD under different insurance schemes in China.

A diversity of studies has assessed the direct medical costs of PD in developed countries [15,20,21,22,23,24,25,26]. A prevalence-based study using a claims database from the US Medicare population reported that the PD-related direct medical costs were USD 3429 in 2018 (USD 3106.5 in 2012) [20]. To estimate the direct medical costs of PD, another US study adopted a prevalence-based approach, using data from the privately insured population and the Medicare-eligible population, and suggested that the direct medical costs per patient were USD 24,439 in 2017 (USD 22,639.6 in 2012) [15]. In Australia, a prospective cohort study from the health system perspective found that the direct medical costs per person amounted to AUD 29,916 in 2012 (USD 19,424.5 in 2012) [21]. In Sweden, an observational, retrospective analysis using Swedish national and regional healthcare data reported that the direct medical costs per patient were SEK 43,114 in 2019 (USD 4323.9 in 2012) [22]. From societal perspectives, a cost-estimation study based on a survey in six European countries found that the direct medical costs per patient were EUR 11,784 in 2008 (USD 18,560.4 in 2012) in Austria; EUR 12,054 in 2008 (USD 18,985.7 in 2012) in Germany; EUR 11,676 in 2008 (USD 18,390.3 in 2012) in Italy; EUR 6612 in 2008 (USD 10,414.2 in 2012) in Czech Republic; EUR 4140 in 2008 (USD 6520.7 in 2012) in Portugal; and EUR 3511 in 2008 (USD 5530.0 in 2012) in Russia [23]. In Hungary, a cross-sectional questionnaire survey conducted in one neurology university clinic discovered that the direct medical costs per capita reached a value of EUR 2149.3 in 2009 (USD 5194.9 in 2012) [24]. In Japan, Yamabe et al. [25] used Japan National Health and Wellness Survey data and found that the direct medical costs attributable to PD were USD 37,994 in 2014 (USD 36,645.5 in 2012). In Singapore, a study conducted by using a prevalence-based bottom-up approach estimated that the direct medical costs per person were SGD 2550 in 2009 (USD 2396.7 in 2012) [26]. Moreover, a few studies in developing countries have also conducted cost-of-illness research on PD patients [27,28]. In the Philippines, a prevalence-based research that used the healthcare system perspective found that the direct medical costs were PHP 67,483.5 in 2020 (USD 3117.7 in 2012) [27]. In Brazil, a retrospective, cross-sectional analysis using a bottom-up approach reported that the direct medical costs per patient were USD 1511.5 in 2017 (USD 1400.2 in 2012) [28].

Only a limited number of studies have evaluated the direct medical costs of PD in China [29,30]. Yang and Chen [29] examined the economic burden of PD through a professional survey including 116 patients with PD from Tianjin Union Medical Center in Tianjin City, and showed that the average annual direct medical costs were USD 1737.93 in 2016 (USD 1640.6 in 2012). Wang et al. [30] analyzed the economic costs of PD by interviewing 190 PD patients who were treated at Ruijin Hospital in Shanghai City and discovered that the average direct medical costs were USD 519 per year per patient in 2004 (USD 612.2 in 2012). Nevertheless, the above China-based studies were limited to one tertiary hospital and their sample sizes were relatively small. In addition, these two studies only included one type of PD patient (IPD patients) excluding SP patients and did not compare the costs of PD patients under different health insurance schemes in China. It was reported that patients with other diseases under different insurance schemes tended to have different costs [31,32,33].

Different from previous research, this study aims to evaluate the direct medical costs of patients with PD with a large sample from an entire city and to identity the potential factors correlated with their inpatient costs using two urban health insurance claims data in Guangzhou City, Southern China.

## 2. Materials and Methods

### 2.1. Data Source

This study used the UEBMI and URBMI administrative claims databases from Guangzhou City, China during the period from 2008 to 2012. Due to the administrative restrictions on accessing the social insurance claims database, this was the most recent data available for this study. The UEBMI and URBMI are two social health insurance schemes covering the population of urban employees and urban non-employed residents in Guangzhou City, with a great deal of differences in service coverages and reimbursement rates [34,35]. The detailed comparisons of UEBMI and URBMI policies for PD patients in Guangzhou City were summarized in Table 1. Until 2012, 93.4% citizens in Guangzhou City were covered by these two urban health insurance schemes [36]. As the capital of Guangdong province, Guangzhou City is one of the largest and most developed cities in Southern China, and thus the sample represents the urban population in Southern China. Information on each PD patient was collected, including sociodemographic characteristics (gender, age, insurance types), medical conditions (primary diagnosis, ICD codes, disease subtypes and comorbidities), and direct medical costs of both outpatient and inpatient care according to actual amounts paid to healthcare providers.

### 2.2. Study Design

A retrospective cross-sectional study was conducted to evaluate the direct medical costs of PD patients using a prevalence-based approach. This study collected all the reimbursement claims submitted for hospitalization care from 1 January 2008 to 31 December 2012 with the primary diagnosis of PD according to the International Classification of Diseases codes, tenth version (ICD-10), including IPD (G20) subtype and SP (G21) subtype [9]. Their outpatient records during the same period were then merged from the outpatient claims database by unique identity number. In total, 2660 PD patients were identified with 2448 UEBMI enrollees and 212 URBMI enrollees.

### 2.3. Cost Estimation and Its Predictors

Information on the direct medical costs of PD patients, including both outpatient and inpatient costs, was derived from the administrative claims databases. The direct medical costs, inpatient costs and outpatient costs all contained the individual out-of-pocket (OOP) spending and the reimbursement part from the UEBMI or URBMI scheme. According to the classification of cost composition used in these two schemes, direct medical costs were divided into five groups: bed fees, medication costs, laboratory and diagnostic costs, non-medication treatment costs and other fees [35,37]. Table 2 described the definitions of different classifications of direct medical costs and showed the calculation methods in detail. All costs were inflation-adjusted to the year 2012 Chinese Yuan (CNY) by using the urban residents’ Consumer Price Index (CPI) of Guangzhou City [36]. The currency exchange rate between US dollars and Chinese Yuan was: USD 1.0 = CNY 6.3125 in 2012. 

As the theoretical framework in the field of healthcare services utilization, Andersen’s behavioral model of health services use [38] was developed in 1968 by Ronald Max Andersen and is widely used for analyzing the influential factors of health behaviors, including healthcare costs [39,40]. In this study, the predictors of inpatient costs for patients with PD were selected based on this theoretical framework [38] and the results of the literature review. Individual influential factors were selected from the following three parts: (1) predisposing characteristics: existing conditions that predispose individuals to health services utilization (such as gender [41,42] and age [28,30,43,44,45,46,47]); (2) enabling characteristics: conditions that facilitate or impede health services usage (for example, type of insurance [31,32]); (3) need characteristics: conditions that medical providers consider as requiring professional treatment (such as disease subtypes [35,37], hospital levels [34,48], length of stay (LOS) [35,48] and presence of any comorbidities [44,49]).

### 2.4. Measures and Variables

In this study, the dependent variable was inpatient costs per patient. The primary independent variable was the type of insurance (patients covered by the scheme of UEBMI or by the URBMI scheme). It did not have a clinically meaningful relationship with other demographic and health variables (gender, age, disease subtypes and comorbidities).

The other covariates included gender, age, disease subtypes, hospital levels, LOS, and presence of comorbidities. Gender was dichotomized as male and female. Age was divided into four subgroups: 18–59 years old, 60–74 years old, 75–89 years old and above 90 years old. Disease subtypes were classified as IPD and SP. Hospitals were marked as three levels in China: primary hospitals, secondary hospitals and tertiary hospitals [16]. In China, according to the bed number, functional orientation and location, hospitals were classified into three different levels [16]. Primary hospitals are often located in communities and provide basic health services to residents for common diseases with fewer than 100 beds [16,48]. Secondary hospitals offer comprehensive health services to several communities and take the charge of teaching and local-based research with 100–500 beds [16,48]. Tertiary hospitals undertake complex health care for several districts and advanced medical training and research with over 500 beds [16,48]. LOS was classified into three groups: less than 15 days, 15–30 days and 30 days and longer. Presence of comorbidities was measured by binary variables indicating whether individuals had the following doctor-diagnosed major chronic diseases: hypertension [50,51,52], diabetes [50,52], coronary heart disease [50], Alzheimer’s disease, schizophrenia [53,54] and mood disorders [52,55]. The detailed measures of variables are shown in Table 3.

### 2.5. Statistical Analysis

This study used frequency and percentage to describe categorical variables, and mean, standard deviation (SD), median and 25th–75th percentiles for continuous variables. Descriptive analysis was used to report the characteristics of patients for the entire sample and two insurance-type subgroups. We estimated the cost composition overall and by types of insurance. Due to the skewed distribution of medical costs, the Kruskal–Wallis rank-sum test was performed to identify whether there were differences in cost compositions between the two insurance schemes. We compared patients’ characteristics between the two insurance subgroups. Friedman’s two-way nonparametric analysis of variance (ANOVA) test was applied to find out the differences in patient characteristics related to inpatient medical costs by type of insurance. In this study, the extension of the generalized linear model (GLM), the extended estimating equations (EEE) model [56], was selected to investigate the potential influential factors of inpatient costs for PD patients. The traditional GLM model often has difficulties in choosing the proper link function and distribution. However, the EEE model can estimate by flexible link and variance functions, which would reduce bias and inefficiency in estimation [56]. The EEE model has been used in previous studies [35,37,57,58,59]. Statistical analyses were conducted using the R language version 4.0.3 and STATA version 12.0 (STATA Corporation, Collection Station, TX, USA).

### 2.6. Ethical Consideration

Ethical approval was obtained from the Institutional Review Board of the School of Public Health, Sun Yat-sen University (Approval No. 2017012).

## 3. Results

### 3.1. Patient Characteristics

A total of 2660 patients diagnosed with PD were identified in this study, of whom 92.0% were covered by the UEBMI scheme and 8.0% covered by the URBMI scheme (see Table 4). Males made up half of the overall sample (50.5%). Males accounted for a larger proportion in the UEBMI subgroup (51.9%), while under the URBMI scheme more than half of the patients were female (66.0%). Overall, the mean age was 71.4 years old (SD = 9.9). Among the UEBMI subgroup, patients aged 75–89 years old (44.1%) outnumbered the other age groups, but patients aged 60–74 accounted for a larger proportion (46.2%) in the URBMI subgroup. Of the total sample, 50.4% were IPD patients, while the rest (49.6%) were patients with SP subtype. The majority of patients (77.1%) received their medical treatment from tertiary hospitals, and 19.0% of patients chose secondary hospitals. The average LOS was 20.0 days (SD = 26.3). A large proportion (54.8%) of patients with PD stayed at hospitals for less than 15 days. Among the total sample, 35.6% of PD patients had hypertension, 11.5% had diabetes, 6.7% had coronary heart disease, 2.6% had Alzheimer’s disease, 0.3% had schizophrenia and 2.6% had mood disorders.

### 3.2. Direct Medical Costs and Costs Composition by Insurance Types

**Figure 1:**
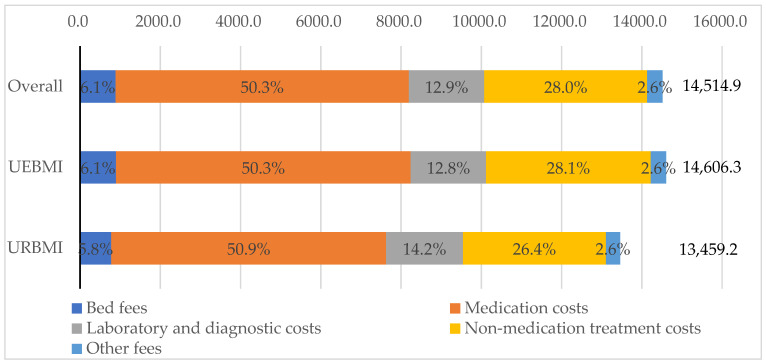
Composition of direct medical costs of Parkinson’s disease patients. Notes: All costs were reported in 2012 Chinese Yuan (CNY). UEBMI—Urban Employee-based Basic Medical Insurance; URBMI—Urban Resident-based Basic Medical Insurance.

For all study population, the direct medical costs per patient with PD were CNY 14,514.9 (USD 2299.4, in 2012), including CNY 13,551.4 (USD 2146.8) for inpatient costs and CNY 963.5 (USD 152.6) for outpatient costs (see Table 5). The inpatient costs accounted for the majority of direct medical costs for PD patients. The proportion of OOP spending out of outpatient costs (57.9%) was much higher than the OOP proportion out of inpatient costs (26.0%). In terms of cost composition, medication costs accounted for 50.3%, followed by non-medication treatment costs (28.0%), laboratory and diagnostic costs (12.9%), bed fees (6.1%) and other fees (2.6%) (see Figure 1).

Table 5 also compared the costs and composition between two different types of insurance. The direct medical costs for PD patients insured by the UEBMI scheme were higher than those covered by the URBMI scheme (CNY 14,606.3 vs. CNY 13,459.2). However, the percentage of OOP spending out of direct medical costs (26.4%) for the UEBMI subgroup was only half of the OOP proportion (50.1%) for the URBMI subgroup, suggesting differences in benefit packages and reimbursement rates between these two types of insurance. With regards to cost composition, medication costs remained the largest percentage of direct medical costs for both the UEBMI subgroup (50.3%) and the URBMI subgroup (50.9%) (see Figure 1). Among direct medical costs, the inpatient costs of PD patients (CNY 13,651.0) under the UEBMI scheme were significantly higher than those of patients (CNY 12,402.2) in the URBMI subgroup (*p* < 0.05). However, the outpatient costs for the UEBMI scheme patients (CNY 955.3) were significantly lower than that of PD patients (CNY 1057.0) covered by the URBMI scheme (*p* < 0.05). The proportion of OOP spending out of inpatient and outpatient costs for UEBMI beneficiaries (24.3% and 56.1%) was much lower than those for patients under the URBMI scheme (47.9% and 76.2%). In addition, the UEBMI enrollees had significantly higher bed fees, non-medication treatment costs and other fees than the URBMI enrollees (*p* < 0.05).

### 3.3. Patient Characteristics Related to Inpatient Costs by Types of Insurance

The patients’ characteristics related to inpatient costs for the entire sample, the UEBMI subgroup and the URBMI subgroup are shown in Table 6. Inpatient costs between the UEBMI patients and URBMI patients were significantly different with regards to gender, age groups, disease subtypes, hospital levels and comorbidities (*p* < 0.05). Among the UEBMI subgroup, male patients incurred higher mean inpatient costs than female patients, but male patients had lower inpatient costs in the URBMI subgroup. The UEBMI patients aged over 90 years old had the highest mean inpatient costs (CNY 16,130.0) among all age groups, while the URBMI patients aged 75–90 had the highest inpatient costs (CNY 13,676.5). For the UEBMI enrollees, patients diagnosed with SP (CNY 12,940.9) reported lower mean inpatient costs than patients with IPD (CNY 14,346.2). However, the mean inpatient costs for patients with SP subtype (CNY 13,362.4) were higher than those for patients with IPD subtype (CNY 11,405.1) under the URBMI scheme. In addition, the highest mean inpatient costs were found in UEBMI patients being hospitalized in tertiary hospitals (CNY 14,324.6), while the highest inpatient costs were found in patients staying in secondary hospitals (CNY 12,953.4) for the URBMI subgroup.

### 3.4. Predictors of Inpatient Costs

As shown in Table 7, it was found that the type of insurance, age, hospital levels, LOS and comorbidities (Alzheimer’s disease and mood disorders) were significantly correlated with inpatient costs of PD. Compared with the URBMI patients, the inpatient costs of PD were CNY 888.1 higher for the UEBMI patients (*p* < 0.05), after adjusting for other factors. Compared with the youngest age group (aged 18–59), the inpatient costs for older age groups of PD patients were CNY 1027.0 higher for those aged 60–74, CNY 1738.0 higher for those aged 75–89, and CNY 5660.5 higher for those aged 90 and above (*p* < 0.01). In comparison with patients staying at secondary hospitals, the costs of inpatient care were CNY 3378.7 lower for PD patients staying at primary hospitals (*p* < 0.01), but CNY 5523.9 higher for patients in tertiary hospitals. After controlling for other covariates, inpatient costs were CNY 6451.2 higher for longer LOS group (15 ≤ Days < 30), and CNY 29,804.3 higher for the longest LOS group (Days ≥ 30), compared to an LOS of less than 15 days (*p* < 0.01). In addition, PD patients with Alzheimer’s disease had significantly lower inpatient costs (CNY 1543.6) (*p* < 0.05), but patients with mood disorders incurred significantly higher inpatient costs (CNY 2305.5) (*p* < 0.01).

## 4. Discussion

This was a retrospective cross-sectional study conducted based on a large sample of 2660 patients with PD in Guangzhou City, Southern China. This study found that the direct medical costs per patient with PD were CNY 14,514.9 (USD 2299.4 in 2012), including CNY 13,551.4 (USD 2146.8) for inpatient costs and CNY 963.5 (USD 152.6) for outpatient costs. The medication costs accounted for the largest percentage of direct medical costs (50.3%) for PD patients. Another key finding was that the type of insurance, age, hospital levels, LOS and comorbidities (Alzheimer’s disease and mood disorders) were significantly correlated with the inpatient costs. To the best of our knowledge, this was the first study using a large sample from an entire city’s claims databases to estimate the direct medical costs of PD and compare the spending of patients with PD between two different urban health insurance schemes in China. 

### 4.1. Costs Comparisons with Previous Studies in Other Countries

In comparison with studies conducted in other countries, we found there were great discrepancies in cost-estimation results for PD patients, after converting all the costs into 2012 US dollars by using the purchasing power parity derived from the Organization of Economic Co-operation and Development (OECD) dataset [60]. In this study, the direct medical costs attributed to PD were CNY 14,514.9 (USD 2299.4 in 2012), much lower than the results in the United States [15,20], Australia [21], Sweden [22], six European countries (Germany, Italy, Austria, Czech Republic, Portugal and Russia) [23], Hungary [24], Japan [25] and the Philippines [27]. However, this figure came close to that of Singapore [26] and was higher than that of Brazil [28]. Among these studies, Wang et al. [15] reported a much higher value of direct medical costs in the US. These high costs might be explained by two reasons. Spending on non-acute institutional care was included when estimating direct medical costs [15]. Moreover, the study population of this US study was different from other studies, consisting of those diagnosed and undiagnosed PD patients by using the more inclusive list of diagnosis codes. In addition, a systematic review study on PD suggested that costs were hardly comparable across different countries, since various methodologies were adopted [61]. For the literature compared in this study, there were differences in the sources of data (claims data vs. survey data), study design (cross-sectional or cohort study), perspective (prospective vs. retrospective) and classification of costs. The discrepancies in costs might be also due to various health care systems between countries.

### 4.2. Costs Comparisons with Previous Studies in China

Compared with prior studies conducted in China [29,30], the present study showed higher direct medical costs of PD. The average direct medical costs of patients with PD were found to be USD 1737.93 in 2015 (USD 1640.6 in 2012) in Yang et al.’s study [29], while another study conducted in Shanghai reported the mean annual direct medical costs of PD to be USD 519 in 2004 (USD 612.17 in 2012) [30]. There were several possible explanations. First, the prior two studies in China only included patients with IPD subtype, whereas patients with IPD and SP subtypes were all included in this research. It was considered that adopting a more inclusive list of diagnosis codes could give a more realistic estimate of the PD costs [15]. Secondly, since these two studies [29,30] selected samples by interviewing patients, patients in a severe disease condition with higher expenses might be excluded. Moreover, both studies collected cost data from only one hospital, while cost estimates in this research were derived from all hospitals in an entire city. Therefore, the direct medical costs of patients with PD reported in our study could be much higher than these two previous China-based studies.

### 4.3. Comparisons of Cost Composition

In terms of cost composition, medication costs accounted for the largest percentage of direct medical costs for PD patients (50.3%) in this study. This proportion of medication costs was close to that of the 49.1% found in Germany [23] and 50.4% in Singapore [26], but higher than the figure in Russia (28%) [23], US (16.3%) [15] and Australia (14.2%) [21]. The variations in medication costs might be due to differences in treatment patterns across countries [23]. The type of drug used could also account for different medication costs of PD [29]. Thus, different preferences in drug use among different countries resulted in cost variations [23]. Levodopa was the main antiparkinsonian medicine widely used by PD patients in China [29]. Furthermore, differences in unit costs and taxes across countries could also partly explain variations in drug costs [23]. Nevertheless, comparisons of medication costs among countries were limited by different pharmaceutical policies, estimation methods and cost components. In China, Yang et al. [29] also discovered that the cost of medication was the main driver, with a share of 34.9%, while another Chinese study found that the proportion of drug costs was particularly high (62.2%) in 2004 [30]. The percentage of medication fees in this study was between the previous two China-based studies. This may be due to the differences in categories of direct medical costs in these studies. Another possible reason is that pramipexole, an expensive dopamine agonist for patients with PD, was included in 2009 China Catalogue of Drugs for Basic National Medical Insurance [62], which resulted in the reduction of its unit price.

### 4.4. Difference in Costs between Two Insurance Schemes

Compared with prior China-based research, this was the first time differences in direct medical costs of patient with PD under the two types of health insurance schemes in China were examined. The UEBMI and URBMI were the two main social health insurance schemes administered by the Chinese government for urban residents only [17]. The inpatient costs of PD patients (CNY 13,651.0) under the UEBMI scheme were significantly higher than those of patients (CNY 12,402.2) in the URBMI subgroup, but the outpatient costs for the UEBMI scheme patients (CNY 955.3) were significantly lower than those of PD patients (CNY 1057.0) covered by the URBMI scheme. In addition, the proportion of OOP spending out of inpatient and outpatient costs for UEBMI beneficiaries (24.3% and 56.1%) was much lower than those for patients under the URBMI scheme (47.9% and 76.2%). The regression analysis also found that the type of health insurance (UEBMI) was significantly correlated with higher inpatient costs of PD patients. These findings were consistent with prior studies exploring disparities in medical costs of patients with other diseases between these two types of health insurance schemes in China [34,35,48]. The possible explanations were discussed as follows. Firstly, UEBMI enrollees had stable jobs and higher incomes and educational levels than URBMI enrollees, and were more likely to pay attention to their health [63]. As a result, patients with higher socioeconomic status covered by the UEBMI scheme were more willing to pay for additional services not covered by the insurance, which may lead to higher expenditures [63]. Secondly, a more comprehensive benefits package including higher reimbursement rates and higher reimbursed ceiling would encourage UEBMI patients to consume more health services and incur higher expenses [48,64]. Thirdly, it was reported that UEBMI beneficiaries demanded more hospital services than they needed, and patients under the UEBMI scheme were more likely to use inpatient services than those covered by the URBMI scheme [65]. Fourthly, since different therapeutic schedules could be adopted by health providers according to patients’ insurance type, the UEBMI enrollees had a higher percentage of inpatients receiving expensive medications than the URBMI enrollees [31,66]. Therefore, the direct medical costs of patients with PD varied between these two health insurance schemes. In order to reduce the inequality caused by the insurance types, efforts should be made to improve and consolidate the fragmented health insurance system in China.

### 4.5. Influential Factors of Inpatient Costs

Disease subtype. This research was the first study from China examining the differences in direct medical costs between two disease subtypes of PD, namely IPD and SP. In the UEBMI subgroup, patients diagnosed with SP (CNY 12,940.9) reported lower inpatient costs than patients with IPD (CNY 14,346.2), while the inpatient costs for patients with SP subtype (CNY 13,362.4) were higher than those for patients with IPD subtype (CNY 11,405.1) in the URBMI subgroup. However, after further controlling for other covariates, the regression analysis indicated that the subtype of disease was not a significant predictor of the inpatient costs of PD. For patients with IPD subtype, the mainstay of therapeutic management was symptomatic treatment with medications that increase dopamine concentrations or directly stimulate dopamine receptors [5]. Apart from medication, surgery treatment options such as deep-brain stimulation (DBS) were also introduced for IPD patients to increase functional independence and quality of life [67,68]. Patients receiving DBS would have higher inpatient costs, due to the expensive fees of DBS procedures and any follow-ups required, plus greater surveillance by healthcare professionals following DBS [69]. Regarding patients with SP subtype, there were many other secondary causes for parkinsonism which may induce different levels of expenditures [7]. The most common form of SP was drug-induced parkinsonism [7], and cessation of the offending drugs was the main treatment for it [8] that might be cheaper. However, the second most frequent cause for SP was vascular parkinsonism, the treatments of which include more expensive dopaminergic replacement therapy, lumbar puncture and the reduction of cardiovascular risk factors [7]. As a result, it was difficult to compare the medical costs of patients among these PD subtypes, since various forms of parkinsonism required totally different therapeutic management.

Age. In accordance with existing literature [45], this study found that age was a significant influential factor on inpatient costs of patients with PD. Compared with the youngest age group, patients in older age groups had significantly higher inpatient costs. Age was the greatest risk factor for IPD patients, and this was mainly due to the natural course of decline in dopaminergic neurons with increasing age [9,67]. For patients with SP subtype, the decreased dopaminergic neurons in the substantial nigra with increasing age would enlarge the susceptibility for additional insults to the dopaminergic system, such as drug and cardiovascular risk factors [70]. 

Hospital level. Consistent with previous research in China [34,35,37], patients staying at higher-level tertiary hospitals had significantly higher inpatient costs. There are three reasons for this finding: First, patients in good financial condition are more willing and able to pursue better medical services in tertiary hospitals, and thus incur higher costs [71,72]. Second, patients with more severe and difficult conditions tend to be treated in tertiary hospitals [16]. Since tertiary hospitals are top-level hospitals in China, the professional level of doctors in tertiary hospitals is generally higher, and more advanced professional skills may result in higher medical expenditures [73]. Third, patients in tertiary hospitals have higher probability to be prescribed expensive medicines that are not included in the Catalogue of Drugs for Basic National Medical Insurance [48,66], leading to higher spending in tertiary hospitals. 

Comorbidities. In this study, comorbidities such as mood disorders and Alzheimer’s disease in PD patients were significantly correlated with variations in inpatient costs. Mood disorders often occurred in PD patients, and psychiatric aspects of PD were associated with numerous adverse outcomes [74] which may lead to higher costs. A study also found that all costs were higher among patients with PD psychosis compared with those with PD without psychosis [75]. Additionally, Alzheimer’s disease and PD were the two most common neurodegenerative disorders [76]. Although pharmacotherapies could slow aspects of cognitive impairment in both Alzheimer’s disease and PD, the benefits were usually marginal and nonsustained [76]. 

LOS. Consistent with previous studies [34,35,37], it was found that a longer LOS could incur higher medical costs of hospitalization, and the mean LOS for patients with PD was 20.0 days. This figure was longer than that reported in Australia (13.5 days) [21], Russia (13.5 days) [45] and Germany (18.0 days) [77]. Since PD resulted in greater health care use, impaired quality of life, disability and caregiver burden, many people with PD needed long-term care (LTC) services [78]. Moreover, PD was the strongest determinant of high formal LTC provision, and overall high care needs could induce high formal LTC provision [79]. In China, given the changing demographic structures, informal care has been the primary source of care for disabled older people [80]. However, the traditional LTC system which heavily relied on informal family care was not sustainable [81]. As a result, the Chinese government launched the long-term care insurance (LTCI) pilot program in 15 pioneer cities of China [82]. Although substantial progress has been made, the coverage of this LTCI scheme for the disabled elderly was still limited due to the strict eligibility criteria [81]. Feng et al. [83] indicated that the introduction of LTCI in Shanghai had significantly reduced the LOS, hospitalization costs, and medical insurance expenditures in tertiary hospitals. Thus, we suggest providing support for the development of a comprehensive LTCI system in China. Furthermore, telemedicine was found to be a sustainable and efficient care delivery method for PD patients living in continuing care institutions, and cost savings could be achieved [84]. Therefore, it was proposed to use telemedicine for the treatment of patients with PD in China.

### 4.6. Limitations

This research had some limitations. First, our study sample only included urban PD patients covered by the two urban health insurance schemes in one city of China and excluded rural residents, from which our findings may not be generalized to the whole population in China and might cause selection bias. Additionally, the present research only evaluated the direct medical costs of patients with PD and did not examine the indirect costs induced by informal care and loss of productivity, which were not included in our insurance claims dataset. As a result, this study was likely to underestimate the total costs of PD in China. Moreover, the clinical severity of PD, which is usually assessed by the Unified Parkinson’s Disease Rating Scale, was also an important influential factor for direct medical costs [85]. However, we cannot include this information in the analysis since this kind of data was not available in our insurance claims dataset. Since having administrative restrictions, we can only access urban claims databases from between 2008 to 2012. Data in this study were a little bit old, which might not show the present direct medical costs of PD patients. In future studies, we will use more recent data to evaluate the direct medical costs of PD. Because data were not available, we could not obtain information on people with PD who were uninsured or purchased private insurance, and thus selection bias might occur. Finally, only six common comorbidities were included, which might cause measurement bias.

### 4.7. Practical Implications

Investigating the direct medical cost of PD and its influential factors has some practical implications. This cost-of-illness study showed real-world data for policymakers and provided guidance to the allocation of health resources and health insurance funds. The cost findings could also be used to conduct cost-effectiveness studies on different treatments for PD patients. Due to budget constraints and resource scarcity in all countries, research analyzing costs, their drivers and the size of costs per patient are important as they provide knowledge to decision-makers at all levels of health care.

## 5. Conclusions

The direct medical costs of patients with PD in China were high compared to the GDP per capita in Guangzhou City and different between the two evaluated types of insurance. Patients with the UEBMI scheme, of older age, with comorbidities, staying in tertiary hospitals and with longer LOS had significantly higher inpatient costs. Thus, policymakers need to reduce the gaps between the two urban insurance schemes in benefit levels, provide support for the development of a comprehensive long-term care insurance system and promote the use of telemedicine in China to reduce the economic burden attributable to PD.

## Figures and Tables

**Table 1:** Comparisons of UEBMI and URBMI policies for Parkinson’s disease in Guangzhou in 2012.

	UEBMI		URBMI	
Inception year	2002		2008	
Eligible population	Urban employed workers (including employees and the retired)		Urban unemployed residents (including children, students, unemployed adults and elderly residents not covered by UEBMI)	
Financing sources	Contributor (8% of annual wage, 6% from employers and 2% from employees)		Government subsidy (70%) Individual premium (30%)	
Accounts	Medical Savings Account (employee contributions and 30% of employer contributions) for outpatient care; Social Risk-Pooling Account (70% of employer contribution) for inpatient care and critical (i.e., chronic or fatal diseases including PD) outpatient care		Social Risk-Pooling Account(all funds) for inpatient care and critical (i.e., chronic or fatal diseases including PD) outpatient care	
	Employees		Children and students	
Deductible (inpatient care)	Primary hospital	CNY 400	Primary hospital	CNY 120
	Secondary hospital	CNY 800	Secondary hospital	CNY 240
	Tertiary hospital	CNY 1600	Tertiary hospital	CNY 480
	The retired		Unemployed adults and elderly residents	
	Primary hospital	CNY 280	Primary hospital	CNY 280
	Secondary hospital	CNY 560	Secondary hospital	CNY 560
	Tertiary hospital	CNY 1120	Tertiary hospital	CNY 1120
	Employees		Children and students	
	Primary hospital	90%	Primary hospital	85%
Reimbursed rate * (inpatient care)	Secondary hospital	85%	Secondary hospital	75%
	Tertiary hospital	80%	Tertiary hospital	65%
	The retired		Unemployed adults and elderly residents	
	Primary hospital	93%	Primary hospital	75%
	Secondary hospital	89.50%	Secondary hospital	65%
	Tertiary hospital	86%	Tertiary hospital	55%
Reimbursed ceiling (inpatient care)	Six times local employees’ annual average wage(CNY 295,680)(in 2012)		Six times local household disposable income(CNY 206,628)(in 2012)	
Deductible (outpatient care)	CNY 0		CNY 0	
Reimbursed rate * (outpatient care)	Community health centers 85% Non-community institutes 65%		Community health centers 85% Non-community institutes 65%	
Reimbursed ceiling (outpatient care)	CNY 150 per person per month		CNY 100 per person per month	

Notes: Information on insurance policies was collected from Guangzhou Statistical bulletin; UEBMI, Urban Employee-based Basic Medical Insurance scheme; URBMI—Urban Resident-based Basic Medical Insurance scheme; CNY—Chinese Yuan; * The percentages were the reimbursed rates that could be reimbursed from Social Risk-Pooling Account in Guangzhou City.

**Table 2:** Definitions of different classifications of direct medical costs.

Costs	Definition	Calculation Equations
Direct medical costs (Type of health services use perspective)	Direct medical costs refer to the annual average medical costs per patient with PD, including outpatient costs and inpatient costs.	=Outpatient costs + Inpatient costs
Outpatient costs	Outpatient costs are the annual average costs for outpatient services.	
Inpatient costs	Inpatient costs are the annual average costs for inpatient services.	
Direct medical costs (Payer perspective)		=Individual out-of-pocket spending + Reimbursement
Individual out-of-pocket spending	Individual out-of-pocket spending is costs that the insurance scheme does not cover and that the individual must pay on their own.	
Reimbursement	Reimbursement is costs reimbursed by the health insurance scheme.	
Direct medical costs (Cost composition perspective)		=Bed fees + Medication costs + Laboratory and diagnostic costs + Non-medication costs + other fees
Bed fees	Bed fees are spending on accommodation when hospitalized.	
Medication costs	Medication costs consist of traditional Chinese medicine and Western medicine expenses.	
Laboratory and diagnostic costs	Laboratory and diagnostic costs are spending on biochemical tests and physical examinations.	
Non-medication treatment costs	Non-medication treatment costs are the costs of blood transfusion, surgery and the other forms of treatment, excluding drug therapy.	
Other fees	Other fees are expenses on other services, such as air conditioning.	

**Table 3:** Variables and measures.

Variables	Measures
Healthcare services use (Dependent variable)	
Inpatient costs	Inpatient costs are the annual average costs for inpatient services, including bed fees, medication costs, laboratory and diagnostic costs, non-medication costs and other fees.
Individual characteristics (Independent variables)	
Predisposing characteristics	
Gender	Female = 0, Male = 1
Age	18 ≤ age < 60, 60 ≤ age < 75, 75 ≤ age < 90, age ≥ 90
Enabling characteristics	
Insurance types	URBMI = 0, UEBMI = 1
Need characteristics	
Disease subtypes	IPD = 0, SP = 1
Hospital level	Primary hospital = 1, Secondary hospital = 2, Tertiary hospital = 3
LOS	Days < 15, 15 ≤ Days < 30, Days ≥ 30
Comorbidities	None, Hypertension, Diabetes, Coronary heart disease, Alzheimer’s disease, Schizophrenia, Mood disorders

Notes: URBMI—Urban Resident-based Basic Medical Insurance scheme; UEBMI—Urban Employee-based Basic Medical Insurance scheme; IPD—Idiopathic Parkinson’s disease; SP—Secondary Parkinsonism; LOS—length of stay.

**Table 4:** Parkinson’s disease patient characteristics.

	Overall	UEBMI	URBMI
No. Patients	2660	2448	212
Gender, n(%)			
Female	1318.0 (49.5)	1178.0 (48.1)	140.0 (66.0)
Male	1342.0 (50.5)	1270.0 (51.9)	72.0 (34.0)
Age (years)			
Mean ± SD	71.4 ± 9.9	71.5 ± 9.8	70.5 ± 10.3
Median (25th–75th)	73.0 (65.0–78.0)	73.0 (66.0–78.0)	71.0 (63.0–78.0)
Age groups, n(%)			
18 ≤ age < 60	335.0 (12.6)	302.0 (12.3)	33.0 (15.6)
60 ≤ age < 75	1145.0 (43.0)	1047.0 (42.8)	98.0 (46.2)
75 ≤ age < 90	1160.0 (43.6)	1080.0 (44.1)	80.0 (37.7)
≥90	20.0 (0.8)	19.0 (0.8)	1.0 (0.5)
Insurance types, n(%)			
URBMI	212.0 (8.0)	/	212.0 (100.0)
UEBMI	2448.0 (92.0)	2448.0 (100.0)	/
Disease subtypes, n(%)			
SP	1319.0 (49.6)	1211.0 (49.5)	108.0 (50.9)
IPD	1341.0 (50.4)	1237.0 (50.5)	104.0 (49.1)
Hospital levels, n(%)			
Primary	103.0 (3.9)	94.0 (3.8)	9.0 (4.2)
Secondary	506.0 (19.0)	472.0 (19.3)	34.0 (16.0)
Tertiary	2051.0 (77.1)	1882.0 (76.9)	169.0 (79.7)
Length of stay (days)			
Mean ± SD	20.0 ± 26.3	20.2 ± 26.9	17.8 ± 17.7
Median(25th–75th)	14.0 (10.0–20.0)	14.0 (10.0–20.0)	12.0 (9.0–19.0)
Days < 15, n(%)	1457.0 (54.8)	1325.0 (54.1)	132.0 (62.3)
15 ≤ Days < 30	855.0 (32.1)	800.0 (32.7)	55.0 (25.9)
Days ≥ 30	348.0 (13.1)	323.0 (13.2)	25.0 (11.8)
Comorbidities, n(%)			
None	1506.0 (56.6)	1349.0 (55.1)	157.0 (74.1)
Hypertension	946.0 (35.6)	900.0 (36.8)	46.0 (21.7)
Diabetes	306.0 (11.5)	296.0 (12.1)	10.0 (4.7)
Coronary heart disease	177.0 (6.7)	168.0 (6.9)	9.0 (4.2)
Alzheimer’s disease	70.0 (2.6)	68.0 (2.8)	2.0 (0.9)
Schizophrenia	9.0 (0.3)	9.0 (0.4)	0.0 (0.0)
Mood disorders	69.0 (2.6)	67.0 (2.7)	2.0 (0.9)
Admission year, n(%)			
Year 2008	364.0 (13.7)	351.0 (14.3)	13.0 (6.1)
Year 2009	419.0 (15.8)	382.0 (15.6)	37.0 (17.5)
Year 2010	440.0 (16.5)	409.0 (16.7)	31.0 (14.6)
Year 2011	652.0 (24.5)	598.0 (24.4)	54.0 (25.5)
Year 2012	785.0 (29.5)	708.0 (28.9)	77.0 (36.3)

Notes: n(%) for categorical variables and mean ± standard deviation(SD) or median(25–27th) for continuous variables; URBMI—Urban Resident-based Basic Medical Insurance scheme; UEBMI—Urban Employee-based Basic Medical Insurance scheme; IPD—Idiopathic Parkinson’s disease; SP— Secondary Parkinsonism.

**Table 5:** Direct medical costs per patient by the type of insurance.

Compositions	Overall	UEBMI	URBMI	*p* Value
No. Patients	2660	2448	212	
Direct medical costs				
Mean (CNY)	14,514.9	14,606.3	13,459.2	0.060
SD	12,511.2	12,615.6	11,208.9	
Bed fees				
Percentage of direct medical costs (%)	6.1	6.1	5.8	
Mean (CNY)	887.3	896.6	779.6	0.012
SD	1011.7	1029.3	774.3	
Medication costs				
Percentage of direct medical costs (%)	50.3	50.3	50.9	
Mean (CNY)	7307.3	7347.0	6848.7	0.344
SD	7063.4	7151.1	5953.0	
Laboratory and diagnostic costs				
Percentage of direct medical costs (%)	12.9	12.8	14.2	
Mean (CNY)	1877.2	1873.8	1917.0	0.833
SD	1645.1	1627.3	1842.1	
Non-medication treatment costs				
Percentage of direct medical costs (%)	28.0	28.1	26.4	
Mean (CNY)	4062.0	4105.6	3558.7	0.003
SD	5700.9	5748.0	5111.2	
Other fees				
Percentage of direct medical costs (%)	2.6	2.6	2.6	
Mean (CNY)	381.0	383.2	355.2	0.048
SD	720.6	730.3	598.0	
Out-of-pocket spending				
Percentage of direct medical costs (%)	28.1	26.4	50.1	
Mean (CNY)	4085.3	3854.7	6747.9	<0.001
SD	3234.3	2904.9	5120.7	
Inpatient costs				
No. patients having hospitalization	2660.0 (100.0)	2448.0 (100.0)	212.0 (100.0)	
Mean (CNY)	13,551.4	13,651.0	12,402.2	0.041
SD	12,424.4	12,540.0	10,962.5	
Out-of-pocket spending				
Percentage of inpatient costs (%)	26.0	24.3	47.9	
Mean (CNY)	3527.8	3318.7	5942.2	<0.001
SD	2942.7	2645.4	4648.0	
Outpatient costs				
No. patients visiting outpatient	1432.0 (53.8)	1339.0 (54.7)	93.0 (43.9)	
Mean (CNY)	963.5	955.3	1057.0	0.024
SD	1461.6	1391.4	2112.5	
Out-of-pocket spending				
Percentage of outpatient costs (%)	57.9	56.1	76.2	
Mean (CNY)	557.5	536.0	805.7	0.118
SD	1126.7	1038.8	1848.2	

Notes: *p* values were based on Kruskal—Wallis rank-sum test; All costs were based on a constant 2012 Chinese Yuan (CNY); URBMI—Urban Resident-based Basic Medical Insurance scheme; UEBMI—Urban Employee-based Basic Medical Insurance scheme; CNY—Chinese Yuan; SD—standard deviation.

**Table 6:** Parkinson’s disease patient characteristics associated with inpatient costs.

Patient Characteristics	Overall		UEBMI		URBMI		*p* Value
No. Patients	n = 2660		n = 2448		n = 212		
	Mean	SD	Mean	SD	Mean	SD	
Gender							0.040
Female	13,291.5	11,917.0	13,291.0	11,839.8	13,295.2	12,592.1	
Male	13,806.8	12,902.7	13,984.8	13,152.1	10,665.7	6488.1	
Age groups							0.030
18 ≤ age < 60	11,505.0	8643.7	11,452.6	8532.7	11,984.6	9732.7	
60 ≤ age < 75	13,205.4	12,166.6	13,363.5	12,264.3	11,515.8	10,984.1	
75 ≤ age < 90	14,443.9	13,493.1	14,500.8	13,633.3	13,676.5	11,478.5	
≥90	15,878.5	11,650.0	16,130.0	11,913.4	11,101.4	/	
Disease subtypes							0.040
SP	12,975.4	11,037.9	12,940.9	10,990.5	13,362.4	11,602.7	
IPD	14,118.1	13,631.9	14,346.2	13,859.9	11,405.1	10,216.0	
Hospital levels							<0.001
Primary	9103.1	10,913.9	9097.4	11,344.3	9162.6	4754.9	
Secondary	11,944.7	13,582.5	11,872.0	13,435.2	12,953.4	15,676.9	
Tertiary	14,171.2	12,117.5	14,324.6	12,277.0	12,463.8	10,047.8	
Length of stay (days)							0.160
Days < 15	8794.3	4209.8	8836.6	4260.9	8369.4	3644.6	
15 ≤ Days < 30	13,890.4	7331.3	13,966.3	7455.4	12,786.2	5124.5	
Days ≥ 30	32,636.0	22,708.1	32,619.4	22,932.6	32,850.8	19,990.2	
Comorbidities							<0.001
None	13,675.2	13,500.4	13,775.8	13,659.2	12,810.3	12,055.2	
Hypertension	13,180.0	10,073.1	13,262.7	10,192.9	11,561.3	7237.2	
Diabetes	14,108.4	12,110.7	14,190.7	12,198.0	11,672.8	9308.0	
Coronary heart disease	12,926.6	9070.6	13,020.5	9211.5	11,172.5	5927.0	
Alzheimer’s disease	14,306.8	8758.7	14,390.4	8840.5	11,464.9	6351.9	
Schizophrenia	14,997.9	9377.3	14,997.9	9377.3	/	/	
Mood disorders	14,644.6	11,905.0	14,739.5	12,045.6	11,464.9	6351.9	

Notes: *p* values were based on Friedman’s two-way non-parametric ANOVA test. All costs were based on a constant 2012 Chinese Yuan (CNY); URBMI—Urban Resident-based Basic Medical Insurance scheme; UEBMI—Urban Employee-based Basic Medical Insurance scheme; IPD—Idiopathic Parkinson’s disease; SP—Secondary Parkinsonism; SD—standard deviation.

**Table 7:** Factors associated with direct inpatient costs (EEE model).

Predictors	Overall (n = 2660)			*p* Value
	Coef.	Adjusted Std.err.	Marginal Effect	
Gender				
Female (Reference)				
Male	0.007	0.017	86.9	0.707
Age group				
18 ≤ age < 60 (Reference)				
60 ≤ age < 75	0.077 ***	0.028	1027.0	0.006
75 ≤ age < 90	0.131 ***	0.029	1738.0	<0.001
≥90	0.371 ***	0.130	5660.5	0.004
Insurance types				
URBMI (Reference)				
UEBMI	0.069 **	0.031	888.1	0.027
Disease subtypes				
SP (Reference)				
IPD	0.010	0.017	127.4	0.565
Hospital levels				
Secondary (Reference)				
Primary	−0.283 ***	0.045	−3378.7	<0.001
Tertiary	0.459 ***	0.027	5523.9	<0.001
Length of stay (days)				
Days < 15 (Reference)				
15 ≤ Days < 30	0.454 ***	0.023	6451.2	<0.001
Days ≥ 30	1.497 ***	0.056	29,804.3	<0.001
Comorbidities				
None (Reference)				
Hypertension	−0.01	0.017	−128.9	0.574
Diabetes	0.043	0.026	569.8	0.099
Coronary	−0.004	0.031	−52.3	0.898
Alzheimer’s disease	−0.122 **	0.047	−1543.6	0.010
Schizophrenia	0.087	0.128	1188.7	0.496
Mood disorders	0.164 ***	0.047	2305.5	<0.001
Year				
Year 2008(Reference)				
Year 2009	0.091 **	0.036	1230.0	0.011
Year 2010	0.110 ***	0.031	1493.2	<0.001
Year 2011	0.108 ***	0.029	1454.4	<0.001
Year 2012	0.158 ***	0.028	2143.1	<0.001
λ	0.213 **	0.092		
θ1	0.238 ***	0.026		
θ2	2.547 ***	0.134		

Notes: The extended estimating equations (EEE) model estimates are shown in the table. Adjusted Std. err. are standard errors adjusted for clustering at the patient level; *** *p* < 0.01,** *p* < 0.05; URBMI—Urban Resident-based Basic Medical Insurance scheme; UEBMI—Urban Employee-based Basic Medical Insurance scheme; IPD—Idiopathic Parkinson’s disease; SP—Secondary Parkinsonism; Coef.—coefficient; Std.err—Standard errors.

## Data Availability

The datasets used and analyzed during the current study are available from the corresponding author on reasonable request.
